# Long-Term Glucocorticoid Exposure and Incident Cardiovascular Diseases—The Lifelines Cohort

**DOI:** 10.1210/clinem/dgae081

**Published:** 2024-03-19

**Authors:** Eline S van der Valk, Mostafa Mohseni, Anand M Iyer, Maartje J B van den Hurk, Robin Lengton, Susanne Kuckuck, Vincent L Wester, Pieter J M Leenen, Willem A Dik, Jenny A Visser, Maryam Kavousi, Mina Mirzaian, Sjoerd A A van den Berg, Elisabeth F C van Rossum

**Affiliations:** Obesity Centre CGG, Erasmus MC, University Medical Center Rotterdam, 3000 CA Rotterdam, The Netherlands; Department of Internal Medicine, Division of Endocrinology, Erasmus MC, University Medical Center Rotterdam, 3000 CA Rotterdam, The Netherlands; Obesity Centre CGG, Erasmus MC, University Medical Center Rotterdam, 3000 CA Rotterdam, The Netherlands; Department of Internal Medicine, Division of Endocrinology, Erasmus MC, University Medical Center Rotterdam, 3000 CA Rotterdam, The Netherlands; Obesity Centre CGG, Erasmus MC, University Medical Center Rotterdam, 3000 CA Rotterdam, The Netherlands; Department of Internal Medicine, Division of Endocrinology, Erasmus MC, University Medical Center Rotterdam, 3000 CA Rotterdam, The Netherlands; Obesity Centre CGG, Erasmus MC, University Medical Center Rotterdam, 3000 CA Rotterdam, The Netherlands; Department of Internal Medicine, Division of Endocrinology, Erasmus MC, University Medical Center Rotterdam, 3000 CA Rotterdam, The Netherlands; Obesity Centre CGG, Erasmus MC, University Medical Center Rotterdam, 3000 CA Rotterdam, The Netherlands; Department of Internal Medicine, Division of Endocrinology, Erasmus MC, University Medical Center Rotterdam, 3000 CA Rotterdam, The Netherlands; Obesity Centre CGG, Erasmus MC, University Medical Center Rotterdam, 3000 CA Rotterdam, The Netherlands; Department of Internal Medicine, Division of Endocrinology, Erasmus MC, University Medical Center Rotterdam, 3000 CA Rotterdam, The Netherlands; Department of Internal Medicine, Division of Geriatric Medicine, Erasmus MC, University Medical Center Rotterdam, 3000 CA Rotterdam, The Netherlands; Department of Immunology, Erasmus MC, University Medical Center Rotterdam, 3000 CA Rotterdam, The Netherlands; Department of Immunology, Erasmus MC, University Medical Center Rotterdam, 3000 CA Rotterdam, The Netherlands; Laboratory of Medical Immunology, Erasmus MC, University Medical Center Rotterdam, 3000 CA Rotterdam, The Netherlands; Obesity Centre CGG, Erasmus MC, University Medical Center Rotterdam, 3000 CA Rotterdam, The Netherlands; Department of Internal Medicine, Division of Endocrinology, Erasmus MC, University Medical Center Rotterdam, 3000 CA Rotterdam, The Netherlands; Department of Epidemiology, Erasmus MC, University Medical Center Rotterdam, 3000 CA Rotterdam, The Netherlands; Department of Clinical Chemistry, Erasmus MC, University Medical Center Rotterdam, 3000 CA Rotterdam, The Netherlands; Obesity Centre CGG, Erasmus MC, University Medical Center Rotterdam, 3000 CA Rotterdam, The Netherlands; Department of Clinical Chemistry, Erasmus MC, University Medical Center Rotterdam, 3000 CA Rotterdam, The Netherlands; Obesity Centre CGG, Erasmus MC, University Medical Center Rotterdam, 3000 CA Rotterdam, The Netherlands; Department of Internal Medicine, Division of Endocrinology, Erasmus MC, University Medical Center Rotterdam, 3000 CA Rotterdam, The Netherlands

**Keywords:** hair glucocorticoids, cardiovascular diseases

## Abstract

**Context:**

Long-term glucocorticoid levels in scalp hair (HairGCs), including cortisol and the inactive form cortisone, represent the cumulative systemic exposure to glucocorticoids over months. HairGCs have repeatedly shown associations with cardiometabolic and immune parameters, but longitudinal data are lacking.

**Design:**

We investigated 6341 hair samples of participants from the Lifelines cohort study for cortisol and cortisone levels and associated these to incident cardiovascular diseases (CVD) during 5 to 7 years of follow-up. We computed the odds ratio (OR) of HairGC levels for incident CVD via logistic regression, adjusting for classical cardiovascular risk factors, and performed a sensitivity analysis in subcohorts of participants < 60 years and ≥ 60 years of age. We also associated HairGC levels to immune parameters (total leukocytes and subtypes).

**Results:**

Hair cortisone levels (available in n = 4701) were independently associated with incident CVD (*P* < .001), particularly in younger individuals (multivariate-adjusted OR 4.21, 95% CI 1.91-9.07 per point increase in 10-log cortisone concentration [pg/mg], *P* < .001). All immune parameters except eosinophils were associated with hair cortisone (all multivariate-adjusted *P* < .05).

**Conclusion:**

In this large, prospective cohort study, we found that long-term cortisone levels, measured in scalp hair, represent a relevant and significant predictor for future CVD in younger individuals. These results highlight glucocorticoid action as possible treatment target for CVD prevention, where hair glucocorticoid measurements could help identify individuals that may benefit from such treatments.

The hypothalamic-pituitary-adrenal (HPA)-axis is crucial for homeostasis and survival, as its end products, glucocorticoid hormones, have pleiotropic effects throughout the body and are important regulators of metabolism and immune function. Upon physiological or psychological stress, the HPA-axis is activated starting with release of corticotropin-releasing hormone (CRH) and adrenocorticotropic hormone (ACTH) from the hypothalamus and anterior pituitary, respectively. This subsequently leads to increasing levels of circulating glucocorticoid hormones.

In humans, the main active glucocorticoid hormone is cortisol. Cortisol binds to the glucocorticoid receptor, a nuclear receptor that is present in nearly all cells of the human body (1). Cortisol can be converted to the inactive hormone cortisone, and vice versa, by 11-beta-hydroxysteroid dehydrogenase, which is one of the mechanisms that regulates the net glucocorticoid effect at tissue level (2).

Pathological disturbances in the amounts of circulating glucocorticoids cause a variety of serious clinical symptoms. In case of severe glucocorticoid excess, known as the Cushing syndrome, patients develop features resembling the metabolic syndrome, including abdominal obesity, insulin resistance, and hypertension (3). It has been hypothesized that also in the general population increased glucocorticoid action contributes to metabolic derangements, perhaps to a lesser extent (4). However, historically it has been difficult to establish a clear relation between the levels of circulating glucocorticoid and anthropometric measurements in the general population, and conflicting results were found when glucocorticoid measurements as in blood, saliva, or urine were related to, for example, body mass index (BMI) and waist circumference (WC) (5). However, all these measurements concern short-term or point measurements and may not represent long-term exposure to glucocorticoids. An interesting perspective may come from hair glucocorticoid (HairGC) analyses, including the measurements of both cortisol and its inactive form cortisone in proximal scalp hair. This is a reliable and easily applicable technique that enables retrospective quantification of the cumulative systemic exposure to glucocorticoids over weeks to months (6-8). There is now consistent evidence that long-term HairGC measurements are correlated to measures of obesity such as BMI and WC (9, 10), as well as cardiovascular diseases (CVD) (11-13), but only from cross-sectional studies, as longitudinal data are lacking. Also, early research focused solely on cortisol, but now evidence is mounting that cortisone may show equal or even stronger associations with metabolic parameters such as BMI and WC (9, 14).

For obesity, we have demonstrated in longitudinal analyses that higher HairGC levels are associated with future weight gain (15). There, we also found first evidence that immune parameters, that is, leukocyte counts and interleukin-6 (IL-6) levels, are linked with HairGC levels. This supports evidence from experimental models indicating close links between the stress response and immune system (16-18). It is, however, less clear whether chronically elevated systemic glucocorticoid levels, reflected in HairGC, also translate to an increased risk in cardiovascular endpoints (19). In the current research, our aim was to investigate whether long-term glucocorticoid levels are associated with incident CVD during follow-up. Our secondary aim was to investigate the relation between long-term glucocorticoid levels and cellular immune parameters.

## Methods

### Participants

We analyzed hair samples in a subset of the Lifelines cohort study. Lifelines, established in 2007, is a multidisciplinary prospective population-based cohort study examining in a unique 3-generation design the health and health-related behaviors of 167 729 persons living in the North of the Netherlands. It employs a broad range of investigative procedures in assessing the biomedical, sociodemographic, behavioral, physical, and psychological factors that contribute to the health and disease of the general population, with a special focus on multi-morbidity and complex genetics (20, 21).

In 2014, participants were invited for a second assessment, which took place between 2014 and 2019 and was considered baseline for the current study. At this visit, hair samples were obtained from all participants who gave permission and had at least 3 cm of hair, following standard operation procedures. The sample was cut as closely to the scalp as possible, at the posterior vertex, and subsequently stored in a dark and dry place until it was analyzed. Prior to the HairGC measurements, we performed a power calculation under the assumptions that the expected incidence of CVD would be 3% in the highest cortisol quartile, and 1% in the lowest quartile (based on Manenschijn et al (11)). With alpha set at 0.05 and 1-β set at 0.90, an estimated 1056 participants were required for each quartile. Accounting for an expected response rate of 85% (known from the Lifelines second visit) and for anticipated technical difficulties in the HairGC measurements, we estimated that we required 6000 to 6500 hair samples for the current study.

To enable future associations with genetic data, we selected the 6341 participants from whom hair samples from 2014 as well as genome-wide association study (GWAS) data were available. The follow-up visit was scheduled to take place between October 2019 and April 2020, but eventually this was extended, as site visits were postponed due to COVID-19 measures. For the current research, we used data of participants who had their visit before December 2021. During the COVID-19 pandemic, participants were invited to fill in an additional online questionnaire (COVID questionnaire). This questionnaire specifically concerned questions about health and behavior related to COVID-19, but also involved questions related to chronic diseases and in particular CVD.

For the Lifelines cohort study, written informed consent was provided by participants and study approval was obtained from the Medical Ethics Committee of the University Medical Center Groningen, Groningen, The Netherlands (2007/152). The Lifelines database was created using the open source MOLGENIS software 9.2.3, built on 2022-04-01 (22).

### Hair Glucocorticoid Measurements

We performed glucocorticoid measurements in hair as previously described, using liquid chromatography–tandem mass spectrometry (LC-MS/MS) adapted with minor modifications (6, 23). To ensure qualitatively valid measurements, we excluded hair samples weighing less than 7.5 mg, where there had been a problem in sample preparation, sample matrix, and/or chromatography, or where mass spectrometry yielded no reliable peak, an aberrant ion-ratio, or co-elution. For both hair cortisol and cortisone, the lower limit of quantification in our assay is currently considered 2.5 pg/mg. The minimum signal-to-noise ratio was set at 10.

### Cardiometabolic Measurements

All anthropometric measurements were performed by technicians trained using standard operating protocols. Height (m) and weight (kg), measured without shoes using standard scales, were used to calculate BMI (kg/m^2^). WC was measured while standing upright, using a SECA 201 measurement tape that was placed between the lowest rib and the iliac crest around the bare stomach.

At the third assessment (follow-up visit), participants were asked whether they had developed any new CVD since they filled in the last questionnaire (yes/no). We considered patients who answered “yes” to this question as cases with incident CVD. Also, participants who reported the presence of cardiovascular diseases at any questionnaire that was performed after the scalp hair collection, and who did not report this at the earlier visits, were considered as cases with incident CVD. A detailed overview of the composition of the outcome is provided in Supplementary data 1 (24).

### Routine Laboratory Measurements

According to routine procedure in the Lifelines cohort, blood was collected and analyzed in the clinical chemistry laboratory of the University Medical Center Groningen (20).

### Other Relevant Covariates

By using questionnaires, we obtained self-reported data concerning education level (classified as low/medium/high according to the standardized cutoffs applicable to the Dutch education system, *Standaard onderwijsindeling*); alcohol use based on questions regarding the number of drinking days per week and the number of drinks per occasion (subsequently classified as “Non-drinkers,” “0-1 drinks per day,” “1-2 drinks per day,” or “More than 2 drinks per day”); and past or current smoking (yes/no). Participants were also asked whether they used any corticosteroid-containing drugs in the last 3 months before taking the hair sample (yes/no) and whether they had been diagnosed with diabetes on any of the previous visits (yes/no).

### Statistical Analysis

Analyses were performed using R Studio (Version 4.2.2). HairGC results below the lower limit of quantification were replaced by 2.5/√2 (25), and because of non-normality they were 10-log transformed for all analyses. As the majority of cortisol data were below the lower limit of quantification, we used cortisol as a dichotomous variable in longitudinal analyses (> 2.5 pg/mg or < 2.5 pg/mg). Descriptive statistics of the baseline measurements are reported as median and interquartile range (IQR) for nonparametric data and mean ± SD for parametric data. We investigated via linear regression whether anthropometric data at baseline were associated with hair cortisone and cortisol levels.

First, we used univariate logistic regression to estimate the influence of HairGC levels and classical cardiovascular risk factors on the outcome incident CVD (present/absent).

Next, we used stepwise logistic regression to estimate the influence of HairGC levels on incident CVD. First, age was added to the model, including the interaction term age*HairGC level (Model A). To investigate sex-differences, we performed exploratory analysis in males and females separately, but refrained from further sex-specific analyses due to a lack of statistical power, particularly for males. Also, insufficient data were available on menopausal status of females.

The final analyses were corrected for age, including the interaction term age*HairGC, sex, corticosteroid use, WC, current smoking, systolic blood pressure, high-density lipoprotein (HDL)- and total cholesterol, having a past history of diabetes mellitus, and having a past history of CVD (Model B). Next, we performed separate sensitivity analysis in participants aged < 60 years and aged ≥ 60 years, also correcting for covariates as described in Model B.

Lastly, we investigated whether cellular immune parameters (ie, total leukocytes, neutrophils, lymphocytes, monocytes, and eosinophils) were associated with hair cortisol and cortisone levels (adjusting for age, sex, BMI, and recent corticosteroid use), and whether they were independent predictors for incident CVD by adding them to Model B.

## Results

### Hair Glucocorticoids and Anthropometrics at Baseline

Baseline characteristics of the full cohort from whom hair samples were obtained (n = 6341), are shown in Table 1. In brief, the population was predominantly female (75%), the mean age was 53 years, and the mean BMI was 26 kg/m^2^.

**Table 1.** Baseline characteristics of the study sample, stratified by sex

	Level	Female	Male	*P*	Missing (%)
n		4758	1571		
Hair cortisone levels, pg/mg, median [IQR]^a^		5.19 [4.02, 7.09]	6.72 [5.15, 9.59]	<.001	25.9
Hair cortisol levels (%)^a^	< 2.5 pg/mg	1619 (76.4)	461 (70.9)	.005	56.2
	> 2.5 pg/mg	499 (23.6)	189 (29.1)		
Age, years, mean (SD)		53.21 (10.06)	54.29 (10.88)	<.001	0.2
BMI (kg/m^2^), mean (SD)		26.31 (4.62)	26.46 (3.64)	.239	0.3
Waist circumference (cm), mean (SD)		87.94 (12.13)	96.10 (10.80)	<.001	0.3
Corticosteroid use in past 3 months (%)	no	3505 (80.6)	1197 (85.4)	<.001	9.3
	yes	846 (19.4)	204 (14.6)		
Past or current smoking (%)	no	3670 (84.7)	1153 (82.7)	.087	9.7
	yes	664 (15.3)	241 (17.3)		
Alcohol use, categorized (%)	≤ 1 drink per day	2509 (58.3)	651 (46.7)	<.001	10.1
	1-2 drinks per day	659 (15.3)	405 (29.1)		
	2 drinks per day	137 (3.2)	202 (14.5)		
	nondrinker	999 (23.2)	136 (9.8)		
HbA1c (mmol/mol), mean (SD)		37.25 (4.88)	37.60 (5.33)	.017	1.2
Fasting blood glucose (mmol/L), mean (SD)		5.07 (0.93)	5.36 (0.96)	<.001	1.7
HDL-cholesterol (mmol/L), mean (SD)		1.71 (0.42)	1.38 (0.34)	<.001	1.1
LDL-cholesterol (mmol/L), mean (SD)		3.33 (0.91)	3.45 (0.90)	<.001	1.1
Systolic blood pressure (mmHg), mean (SD)		128.18 (17.12)	133.44 (15.67)	<.001	0.3
Diastolic blood pressure (mmHg), mean (SD)		72.88 (8.93)	78.06 (9.17)	<.001	0.3
Past history of cardiovascular diseases (%)	not mentioned	4577 (96.2)	1450 (92.3)	<.001	0.0
	present	181 (3.8)	121 (7.7)		
Past history of diabetes mellitus (%)	not mentioned	4594 (96.6)	1501 (95.5)	.078	0.0
	present	164 (3.4)	70 (4.5)		
Leukocytes, 10E9/L		6.09 (1.70)	6.16 (2.15)	.210	1.1
Monocytes, 10E9/L		0.48 (0.14)	0.55 (0.16)	<.001	1.9
Lymphocytes, 10E9/L		2.03 (0.62)	1.98 (1.30)	.027	1.9
Neutophilic granulocytes, 10E9/L		3.29 (1.21)	3.30 (1.11)	.676	1.9
Eosinophilic granulocytes, 10E9/L		0.19 (0.13)	0.21 (0.14)	<.001	1.9

Parametric data are presented as mean(SD), nonparametric data are presented as median (IQR; interquartile range).

Abbreviations: BMI, body mass index; HDL, high-density lipoprotein; LDL, low-density lipoprotein.

**Figure 1.**
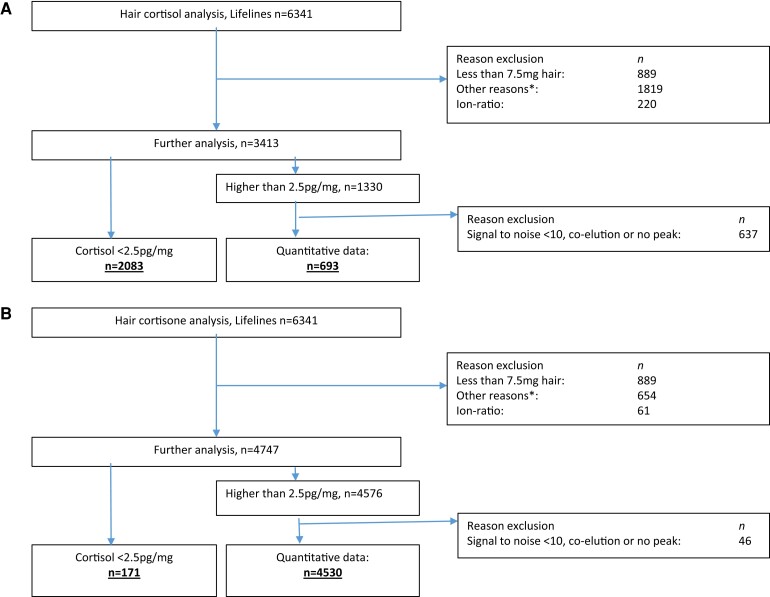
Flowchart of hair cortisol (A) and hair cortisone analysis (B). *Indicates problem with sample preparation, sample matrix, and/or chromatography.

^a^Flowchart of hair cortisol and cortisone results is given in Fig. 1A and 1B.

Hair cortisol levels were below the lower limit of quantification (2.5 pg/mg) in the majority of participants in whom valid measurements could be performed (n = 2083; 33%). In 693 participants (11%), hair cortisol levels were > 2.5 pg/mg. A flowchart of hair cortisol results is provided in Fig. 1A. For cortisone, 171 (2.7%) participants had cortisone levels below the lower limit of quantification. Baseline hair cortisone levels were quantifiable in a total of 4530 participants (71.4%, flowchart provided in Fig. 1B).

In individuals with quantifiable hair cortisol levels (n = 693), there was a positive association between hair cortisol levels and BMI (*r* = 0.099, beta = 1.872, *P* = .0097), and between hair cortisol levels and waist circumference (*r* = 0.109, beta = 5.92, *P* = .004). For cortisone, we found significant baseline associations between hair cortisone and BMI (*r* = 0.075, beta = 1.51, *P* < .00001) and between hair cortisone and WC (*r* = 0.137, beta = 7.67, *P* < .00001).

### Hair Glucocorticoids and Incident Cardiovascular Events During Follow-Up

A total of 4218 participants had follow-up data, from either the follow-up visit and/or the COVID questionnaire or 2B questionnaire (Supplemental data 1 & 2 (24)). A total of 348 individuals reported one or multiple new cardiovascular disease manifestations during follow-up (incident CVD). This was specified by 53 participants as a heart attack (15%), 30 participants specified it as “narrowing of the arteries in the legs” (9%), 30 participants as “stroke” (9%), and 47 reported “heart failure” (14%). The remaining cases were unspecified, mainly because these answers were obtained from questionnaires where a specification was not requested.

In univariate logistic regression analysis (results provided in Supplemental data 3 (24)), baseline hair cortisone levels were significantly associated with incident CVD during follow-up (OR = 3.08 [95% CI, 1.79-5.20] *P* < .001) on a continuous scale as well as in quartiles. Here, hair cortisol levels did not predict incident CVD (*P* = .73), neither when dichotomized (*P* = .30).

### Age-Specific Analysis

**Figure 2.**
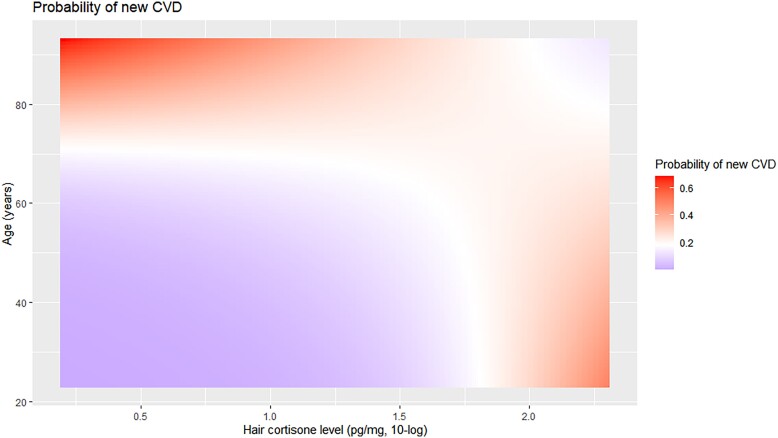
Heatmap showing the predicted probability of cardiovascular diseases during follow-up, according to the interaction between age at baseline and hair cortisone levels (pg/mg, 10-log transformed) at baseline. Red color indicates high probability of new cardiovascular diseases (CVD), purple indicates lower probability.

In a multivariate logistic regression model (Model A), we investigated the association between baseline cortisone levels on the outcome incident CVD, correcting for age and the interaction hair cortisone*age. We found that hair cortisone, age, and the interaction between age and cortisone were all significantly associated with the outcome (*P* = .01, *P* < .001, and *P* = .03, respectively), indicating that having higher hair cortisone levels increases the probability of incident CVD in an age-dependent manner. A heatmap showing the interaction between age and hair cortisone level on the probability of incident CVD is provided in Fig. 2.

### Sex-Specific Analysis

In a separate univariate analysis within female participants only (n = 2441, 165 CVD cases) hair cortisone levels were still significantly associated with CVD (*P* < .001, OR = 3.05 [95% CI, 01.56-5.79]). In males, the association lost significance (n = 704, 78 CVD cases, *P* = .2, OR = 1.81 [95% CI, 0.65-4.91]). Due to lack of power, we refrained from further sex-specific analyses, but we used “sex” as a covariate in all multivariate models.

### Fully Adjusted Models

**Figure 3.**
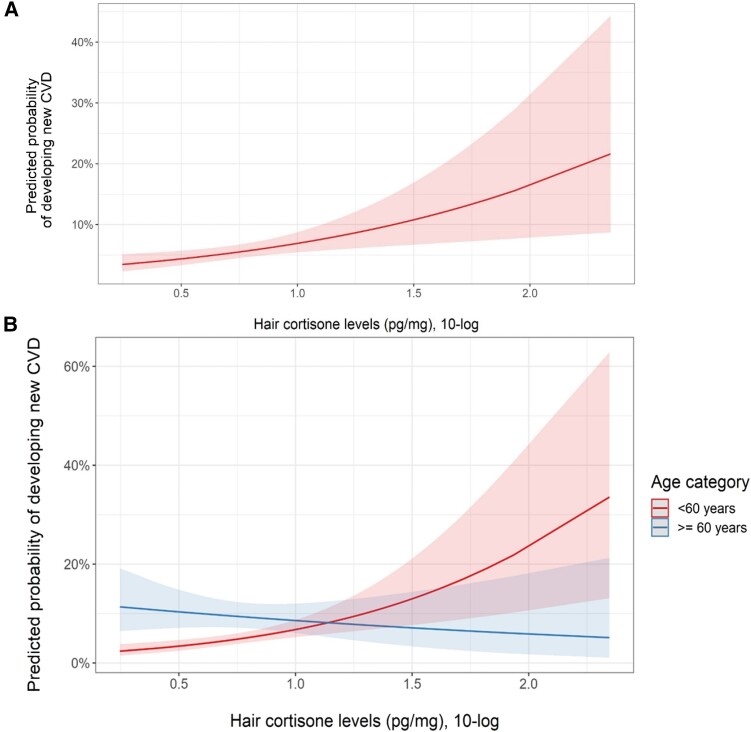
A, Graphical representation of the predicted probability of developing new cardiovascular diseases (CVD) during follow-up, according to baseline hair cortisone levels (pg/mg, 10-log transformed), corrected for age (per 10 years increase), sex, waist circumference, corticosteroid use in the last 3 months, smoking, systolic blood pressure (per 10 mmHg increase), and past medical history of diabetes mellitus, in a logistic regression model with incident CVD as the outcome variable. Red line indicates predicted probability, with red bands indicating 95% CI. B, Graphical representation of the predicted probability of developing new cardiovascular diseases (CVD) during follow-up, according to baseline hair cortisone levels (pg/mg, 10-log transformed), split by age category (< 60 years or ≥ 60 years), including the interaction term age category*hair cortisone. Analyses are corrected for sex, waist circumference, corticosteroid use in the last 3 months, smoking, systolic blood pressure (per 10 mmHg increase), high-density lipoprotein (HDL) cholesterol, total cholesterol, past medical history of diabetes mellitus, and past medical history of CVD, in a logistic regression model with incident CVD as the outcome variable. Red line indicates predicted probability, with red bands indicating 95% CI for the *younger* individuals. Blue line indicates predicted probability for the *elder* individuals, with blue bands indicating 95% CI.

In the fully adjusted model (using Model B, including classical cardiovascular risk factors), hair cortisone was still associated with incident CVD (Table 2A, graphical representation of predicted probability for incident CVD by hair cortisone levels shown in Fig. 3A).

**Table 2.** Results of the multivariate logistic regression for the outcome self-reported incident cardiovascular diseases during the 5 to 7 years of follow-up, for (a) hair cortisone (as continuous predictor, including the interaction term hair cortisone*age) and (b) hair cortisol (dichotomized as above/below the lower limit of quantification [2.5pg/mg])

Characteristic	OR^a^	95% CI^a^	*P* value
***Hair cortisone levels*** *(total n = 2878, number of events = 214)*			
Hair cortisone level (pg/mg), 10-log	83.3	2.36, 2855	.**015**
Age, per 10 years	2.64	1.55, 4.51	**<**.**001**
Hair cortisone level (pg/mg), 10-log * Age, per 10 years	0.52	0.28, 0.96	.**038**
Sex, male	1.07	0.75, 1.51	.7
Waist circumference	1.00	0.99, 1.02	.8
Any corticosteroid use in last 3 months, yes	1.06	0.72, 1.53	.7
Current smoking, yes	0.96	0.60, 1.48	.9
Systolic blood pressure, per 10 mmHg increase	1.19	1.09, 1.30	**<**.**001**
Past history of diabetes mellitus	2.13	1.15, 3.79	.**012**
HDL-cholesterol, mmol/L	0.80	0.53, 1.19	.3
Total cholesterol, mmol/L	0.92	0.79, 1.08	.3
Past history of cardiovascular diseases	3.00	1.75, 4.99	**<**.**001**
***Hair cortisol levels*** *(total n = 1739, number of events = 140)*			
Hair cortisol levels > 2.5 pg/mg	8.34	.79, 84.5	.075
Age, per 10 years	1.80	1.42, 2.28	**<**.**001**
Hair cortisol levels > 2.5 pg/mg * Age, per 10 years	0.69	.46, 1.04	.078
Sex, male	1.08	.69, 1.65	.7
Waist circumference	1.00	.98, 1.01	.7
Any corticosteroid use in last 3 months, yes	0.79	.48, 1.27	.4
Current smoking, yes	1.00	.55, 1.72	>.9
Systolic blood pressure, mmHg	1.01	1.00, 1.03	.**010**
High-density lipoprotein cholesterol, mmol/L	0.61	.35, 1.02	.063
Total cholesterol, mmol/L	0.98	.80, 1.18	.8
Past history of diabetes mellitus	2.07	.94, 4.27	.058
Past history of cardiovascular diseases	3.27	1.64, 6.19	**<**.**001**

Results of a multivariate logistic regression model are provided, where incident cardiovascular diseases in 5-7 years follow-up was used as outcome. Included covariates were age, sex, waist circumference (per cm increase), self-reported corticosteroid use in the past 3 months (yes/no), smoking, systolic blood pressure (odds ratio presented per 10 mmHg increase) and self-reported past history of diabetes mellitus (yes/no). *P* values were significant at *P* < .05 (presented in bold).

^a^Odds ratios (OR) and 95% confidence intervals (95% CI) are presented.

Cortisol was not significantly associated with CVD in Model B when used on a continuous scale (*P* = .193 and *P* = .209 for the interaction term cortisol*age). However, when dichotomized in those below and above the lower limit of quantification, having high hair cortisol (> 2.5 pg/mg) was in trend associated with future CVD (*P* = .072 for hair cortisol, *P* < .001 for age, and *P* = .075 for the interaction term cortisol*age; Table 2B). In the subgroup of participants with known cortisol data, cortisone was still significant in univariate analysis but not in multivariate analyses according to Model A and B (n = 1847, *P* = .02 for univariate analysis; n = 1847 and *P* = .11 for Model A; and n = 1695 and *P* = .2 in Model B). In the subgroup of participants without past CVD, hair cortisone levels were still significantly associated with incident CVD (*P* < .019 for hair cortisone, *P* = .057 for hair cortisone*age when used in Model B).

### Sensitivity Analysis in Age Categories

Within the subgroup of individuals < 60 years, hair cortisone was strongly and significantly associated with incident CVD after adjustment for relevant covariates via Model B (OR = 4.21 [95% CI, 1.91-9.07], *P* = .005, Table 3). In elderly individuals (≥ 60 years), hair cortisone levels were not significantly associated with CVD after adjustment for relevant covariates (Table 3, visual representation in Fig. 3B).

**Table 3.** Results of the sensitivity analyses in subgroups based on age (categorized as < 60 years and ≥ 60 years)

Predicting incident cardiovascular diseases by baseline hair cortisone, split by age category			
Characteristic	OR	95% CI	*P* value
**< 60 years** *(total n = 2229, number of events = 125)*			
Hair cortisone level (pg/mg), 10-log	4.21	1.91, 9.07	**<**.**001**
Sex, male	1.14	.71, 1.79	.6
Waist circumference	1.01	.99, 1.02	.5
Any corticosteroid use in last 3 months, yes	1.13	.68, 1.81	.6
Current smoking, yes	0.92	.52, 1.52	.7
Systolic blood pressure, per 10 mmHg increase	1.24	1.10, 1.40	**<**.**001**
High-density lipoprotein cholesterol, mmol/L	1.12	.66, 1.88	.7
Total cholesterol, mmol/L	0.94	.76, 1.15	.5
Past history of diabetes mellitus	3.31	1.37, 7.24	.**004**
Past history of cardiovascular diseases	3.03	1.32, 6.25	.**005**
**≥ 60 years** (t*otal n = 649, number of events = 89)*			
Hair cortisone level (pg/mg), 10-log	0.67	.23, 1.81	.4
Sex, male	1.07	.61, 1.85	.8
Waist circumference	1.00	.97, 1.02	.7
Any corticosteroid use in last 3 months, yes	0.93	.51, 1.63	.8
Current smoking, yes	0.88	.34, 1.99	.8
Systolic blood pressure, per 10 mmHg increase	1.20	1.05, 1.37	.**006**
High-density lipoprotein cholesterol, mmol/L	0.56	.29, 1.04	.075
Total cholesterol, mmol/L	0.96	.76, 1.22	.7
Past history of diabetes mellitus	1.58	.64, 3.62	.3
Past history of cardiovascular diseases	3.33	1.58, 6.80	.**001**

Results of a multivariate logistic regression are provided, where hair cortisone levels (pg/mg, 10 log transformed), sex, waist circumference (per cm increase), self-reported corticosteroid use in the past 3 months (yes/no), smoking, systolic blood pressure (odds ratio presented per 10 mmHg increase) and self-reported history of diabetes mellitus (yes/no) were used as predictors. Odds ratios (OR) and 95% CI are presented. *P* values were significant at *P* < .05 (presented in bold).

### Immune Parameters at Baseline

Baseline hair cortisone levels were significantly associated with baseline total leukocytes, neutrophils, and monocytes (*r* = 0.065, *r* = 0.076, and *r* = 0.11 respectively; *P* = .0001, *P* = 3E-7, and *P* = 5.3E-14, respectively), and in trend to lymphocytes (*P* = .078) and eosinophils (*P* = .067). After adjustment for age, sex, BMI, and corticosteroid use, hair cortisone was significantly associated with leukocytes, neutrophils, monocytes, and lymphocytes (all *P* < .001, Table 4). Similarly, hair cortisol levels were associated with baseline leukocytes, neutrophils, lymphocytes, and eosinophils (*r* = 0.045, *r* = 0.042, *r* = 0.046, and *r* = 0.038, respectively; *P* = .018, *P* = .03, *P* = .017, and *P* = .047), but not monocytes (*r* = 0.025, *P* = .19). After adjustment for age, sex, BMI, and corticosteroid use, only lymphocytes were significantly associated with hair cortisol (*P* = .04, Table 5).

**Table 4.** Cross-sectional linear regression models investigating the associations between hair cortisone levels and immune parameters, adjusting for age, sex, recent corticosteroid use, and body mass index (BMI, kg/m^2^)

	Total leukocytes (10 e-9/L)		Neutrophil granulocytes (10 e-9/L)		Monocytes (10 e-9/L)		Lymphocytes (10 e-9/L)		Eosinophil granulocytes (10 e-9/L)	
*Predictors*	*Estimates*	*P*	*Estimates*	*P*	*Estimates*	*P*	*Estimates*	*P*	*Estimates*	*P*
Hair cortisone levels (pg/mg), 10-log	0.48 (0.24-0.71)	**<.001**	0.44 (0.28-0.60)	**<.001**	0.04 (0.02-0.06)	**<.001**	0.09 (0.00-0.17)	**.046**	0.00 (−0.02-0.02)	.781
Age, per 10 years	−0.15 (−0.20-−0.10)	**<.001**	−0.14 (−0.18-−0.11)	**<.001**	0.01 (0.00-0.01)	**<.001**	−0.01 (−0.03-0.01)	.363	0.00 (−0.00-0.01	.483
Sex, male	−0.01 (−0.14-0.11)	.821	−0.05 (−0.14-0.04)	.260	0.05 (0.04-0.07)	**<.001**	−0.07 (−0.12-−0.02)	**.003**	0.03 (0.02-0.03)	**<.001**
Recent corticosteroid use, yes	0.27 (0.14-0.40)	**<.001**	0.19 (0.09-0.28)	**<.001**	0.02 (0.01-0.03)	**<.001**	0.04 (−0.01-0.09)	.127	0.03 (0.02-0.04)	**<.001**
BMI (kg/m^2^)	0.07 (0.06-0.08)	**<.001**	0.05 (0.04-0.06)	**<.001**	0.00 (0.00-0.00)	**<.001**	0.02 (0.01-0.02)	**<.001**	0.00 (−0.00-0.00)	.064
Observations	4243		4210		4210		4210		4210	

Results of a multivariate linear regression model where hair cortisone levels (pg/mg), age, sex, recent corticosteroid use and body mass index (BMI) are included. Linear regression estimates and 95% confidence intervals are provided. *P* values were significant at *P* < .05 (presented in bold).

**Table 5.** Cross-sectional linear regression models investigating the associations between hair cortisol levels and immune parameters, adjusting for age, sex, recent corticosteroid use and body mass index (BMI, kg/m^2^)

*Predictors*	Total Leukocytes (10 e-9/L)		Neutrophil Granulocytes (10 e-9/L)		Monocyte (10 e-9/L)		Lymphocytes (10 e-9/L)		Eosinophil Granulocytes (10 e-9/L)	
	*Estimates*	*P*	*Estimates*	*P*	*Estimates*	*P*	*Estimates*	*P*	*Estimates*	*P*
Hair cortisol levels (pg/mg), 10-log	0.36 (−0.04-0.76)	.075	0.22 (−0.05-0.50)	.112	0.01 (−0.02-0.04)	.471	0.15 (0.01-0.30)	**.036**	0.02 (−0.01-0.05)	.158
Age, per 10 years	−0.18 (−0.24-−0.11)	**<.001**	−0.15 (−0.19-−0.10)	**<.001**	0.01 (0.00-0.01)	**.001**	−0.02 (−0.05-0.00)	.058	0.00 (−0.00-0.01)	.849
Sex, male	0.06 (−0.10-0.22)	.499	0.02 (−0.09-0.13)	.659	0.06 (0.05-0.07)	**<.001**	−0.08 (−0.14-−0.03)	**.004**	0.03 (0.01-0.04)	**<.001**
Recent corticosteroid use, yes	0.29 (0.12-0.47)	**.001**	0.21 (0.09-0.33)	**.001**	0.02 (0.01-0.03)	**.005**	0.03 (−0.03-0.10)	.333	0.03 (0.02-0.04)	**<.001**
BMI (kg/m^2^)	0.07 (0.06-0.09)	**<.001**	0.05 (0.04-0.06)	**<.001**	0.00 (0.00-0.00)	**<.001**	0.02 (0.01-0.02)	**<.001**	0.00 (0.00-0.00)	**.030**
Observations	2514		2499		2499		2499		2499	

Results of a multivariate linear regression model where hair cortisol levels (pg/mg), age, sex, recent corticosteroid use and body mass index (BMI) are included. Linear regression estimates and 95% confidence intervals are provided. *P* values were significant at *P* < .05 (presented in bold).

### Immune Parameters at Follow-Up

When total leukocytes, neutrophils, and monocytes were added to Model B with cortisone as predictor, only monocytes were a significant independent risk factor for incident CVD (OR = 3.37 [95% CI, 1.19-9.36], *P* = .021), leukocytes and neutrophils were not significant (Supplemental data 4A & 4B (24)). In the same model with dichotomized cortisol, none of the immune parameters were significant predictors of incident CVD.

## Discussion

We prospectively evaluated the association between baseline hair glucocorticoid levels and incident cardiovascular diseases during 5 to 7 years of follow-up in a large population-based cohort study. Hair cortisone levels showed strong associations with incident CVD, even after adjustment for known predictors of CVD. This effect was driven in particular by the younger individuals, as hair cortisone was highly significantly and relevantly associated with incident CVD in the subcohort of individuals < 60 years of age. The magnitude of the association was comparable to other known risk factors for CVD, such as a history of diabetes mellitus and higher systolic blood pressure. Hair cortisol levels were not significantly, but in trend, associated with incident CVD, perhaps due to lack of statistical power. Hair cortisone and hair cortisol were both associated with multiple cellular immune parameters in cross-sectional analyses; in longitudinal analysis, only monocyte count was an independent predictor of incident CVD.

Although it has been established that higher hair glucocorticoid levels, reflecting chronic systemic exposure, are associated on a cross-sectional level with cardiometabolic parameters, such as BMI, WC, metabolic syndrome, and CVD (9, 11, 14), the relevance of these findings was unsure as longitudinal studies were lacking (4, 19). Our study is now the first to fill this gap, providing evidence on the relevant clinical outcome of incident CVD. This endorses the concept that higher exposure to endogenous glucocorticoid levels, also in individuals without clear clinical endogenous hypercortisolism, may not only represent a risk factor for weight gain (15), but also for clinically relevant endpoints, such as incident CVD. For morning plasma cortisol, it has already been prospectively demonstrated that this was associated with higher risk for CVD (26). However, those odds ratios were much lower than those observed in the current study, indicating that perhaps a measure of cumulative exposure, which is believed to be assessed by hair glucocorticoid analyses, is of higher interest than morning (time point) measurements of cortisol. Importantly, it should be noted that despite the associations that were found, definite evidence for causation is still lacking.

Interestingly, the association seems to apply particularly to incident CVD at a younger age (≤ 60 years at baseline). For traditional cardiovascular risk factors, relative risk contributions are known to decrease with age (27, 28), suggesting a multifactorial etiology, whereas age per se is strongly associated with increased CVD risk. A high a priori risk in older individuals may thus attenuate the relative contribution of other traditional risk factors. Of note, it is well established that mean daily glucocorticoid levels and hair glucocorticoid levels generally increase with aging (10, 29). In this regard, it can be hypothesized that higher glucocorticoid levels in younger individuals may actually reflect underlying accelerated biological aging, where these individuals resemble older individuals with regard to CVD risk. This may be mediated by an acceleration of the age-related increase in activation of the immune system (“inflammaging”), which is highly relevant in the development of atherosclerosis (30). Indeed, in the current work we found significant cross-sectional associations between various cellular immune parameters and glucocorticoid levels. In the multivariate-adjusted longitudinal analyses, monocyte counts were the only cellular immune parameter that was a significant risk factor for CVD, independent of glucocorticoid levels. This indicates tight links between HPA-axis activation and immune activation, *post aut propter* translating to a higher risk for incident CVD. Also, we confirmed earlier findings that monocytes are most closely linked to the development of CVD, which is in line with their pathogenic role (31).

Of note, the current work supports previous findings regarding metabolic syndrome and waist circumference, suggesting that among HairGC, the most pronounced associations with cardiometabolic health are seen for the biologically inactive cortisone, instead of cortisol (9, 14). It has been hypothesized that hair cortisone levels reflect the “reservoir” of circulating glucocorticoids, that can be activated by the enzyme 11-beta-hydroxysteroid dehydrogenase type 1 to biologically active cortisol (4, 9). However, it should be noted that we did not meet the anticipated statistical power that was required to detect a significant difference for cortisol. Thus, our cortisol results should be interpreted with high caution, and the high number of cortisol measurements that had to be excluded for technical reasons is a limitation of the current study.

Another important limitation of our work is that the endpoint CVD has been based on self-reporting of a diagnosis, with several (structured) questionnaires that, however, used slightly different phrasings. It is known that self-reporting of incident diseases has a high specificity, but sensitivity tends to be lower (32). Furthermore, there is a relatively large group of individuals from whom we did not have any follow-up (33%). This subgroup was not different regarding age and sex but had a significantly higher prevalence of all risk factors, including hair cortisone levels. Perhaps part of the attrition is due to increased morbidity or even (cardiovascular) mortality, which is, however, unknown.

Furthermore, inherent in the population of the northern provinces of the Netherlands, the Lifelines cohort includes relatively few ethnic minorities, hampering generalizability to other populations. Also, in line with other population studies using HairGC measurements, we have an overrepresentation of females (15, 33, 34), limiting further sex-specific analyses due to lack of power in males.

Next, we did not have any data on medication use (eg, statins, antihypertensives, and glucose-lowering drugs) which may have induced a bias.

In order to determine the future clinical consequences of our findings, we need to better understand why some individuals have increased systemic glucocorticoid exposure. Possible explanations include increased cortisol production, for example, in response to low-grade inflammation (as suggested by the consistent associations with immune parameters that we found), or following psychological or physical stressors (eg, chronic pain, sleep deficit, endocrine disruptors) (4), and/or altered cortisol metabolism eg, in the liver or visceral adipose tissue (35).

In Cushing disease, treating the hypercortisolism greatly reduces cardiovascular risk (36). However, until now, in people without Cushing disease there were only modest effects of drugs targeting glucocorticoid action (37-39). It can be speculated that HairGC measurements can be used to identify individuals at risk who may benefit from pharmacological or non-pharmacological glucocorticoid-lowering interventions or preventive strategies, ultimately decreasing cardiovascular risk.

In summary, we demonstrate that long-term glucocorticoid levels, specifically cortisone, are associated with an increased risk of incident CVD in younger individuals, even after adjustment for classical risk factors and with comparable effect sizes. Our results thus highlight the potential role of glucocorticoid action in the development of CVD and reveal hair glucocorticoid levels as promising biomarkers that may be used to identify individuals with increased cardiovascular risk.

## Data Availability

Data may be obtained from a third party and are not publicly available. Researchers can apply to use the Lifelines data used in this study. More information about how to request Lifelines data and the conditions of use can be found on their website (https://www.lifelines.nl/researcher/how-to-apply).

## Abbreviations

BMI: body mass index
CVD: cardiovascular disease
HairGC: glucocorticoid levels in scalp hair
HPA: hypothalamic-pituitary-adrenal
IL-: interleukin
IQR: interquartile range
OR: odds ratio
WC: waist circumference
